# Feasibility and acceptability of point-of-care ultrasound delivered by midwives during routine antenatal care in Malawi: a prospective implementation science study

**DOI:** 10.1136/bmjopen-2025-100515

**Published:** 2025-08-10

**Authors:** Chipiliro Payesa, Linly Seyama, Yamikani Chimwaza, Fidelis Sindani, Yankho Kanise, Elida Bvutula, Modesta Phiri, Pempho Nyangulu, Luis Gadama, Fanny Kachale, Gladys Gadama, Mary Mwale, Gayane Yenokyan, Pooja Sripad, Anne Hyre, Lisa M Noguchi, Sufia Dadabhai

**Affiliations:** 1 Johns Hopkins Research Project, Kamuzu University of Health Sciences, Blantyre, Malawi; 2 Jhpiego Malawi, Jhpiego Corporation, Baltimore, Maryland, USA; 3 Blantyre District Health Office, Queen Elizabeth Central Hospital, Blantyre, Malawi; 4 Department of Obstetrics and Gynaecology, Queen Elizabeth Central Hospital, Blantyre, Malawi; 5 Government of Malawi Ministry of Health, Lilongwe, Malawi; 6 Biostatistics, Johns Hopkins University Bloomberg School of Public Health, Baltimore, Maryland, USA; 7 Jhpiego Corporation, Baltimore, Maryland, USA; 8 Epidemiology, JHSPH, Baltimore, Maryland, USA

**Keywords:** Pregnant Women, Ultrasound, Feasibility Studies, Midwifery

## Abstract

**Abstract:**

**Objectives:**

To evaluate the feasibility and acceptability of integrating point-of-care ultrasound scan (POCUS) by midwives into routine antenatal care (ANC) services.

**Design:**

Prospective, observational, multiphase, implementation science study.

**Main outcome measures:**

Primary outcomes included the proportion of midwives who completed training and competency checks for basic obstetric scanning using a POCUS device; the feasibility and acceptability of midwife-delivered POCUS from the perspectives of midwives and pregnant women captured on structured questionnaires; and the proportion of scans meeting predefined quality standards. Secondary outcomes included responses to acceptability-related questionnaires administered to midwives and pregnant women.

**Setting:**

Rural, periurban and urban health centres in Blantyre District, Malawi.

**Participants:**

Pregnant women attending ANC and midwives providing care at participating health facilities.

**Results:**

Obstetric registrars trained and mentored 45 midwives, and 42 (93%) completed the training. Most midwives (95%, n=40) found providing POCUS during ANC was feasible and acceptable. Overall, device durability was rated positively. Of the 1499 pregnant women who received a scan, 99% (n=1484) reported that receiving an ultrasound from a midwife during ANC was acceptable. Independent assessors determined that over 70% of the subsample of reviewed scans met minimum quality standards.

**Conclusions:**

Midwife-delivered POCUS is feasible and highly acceptable in diverse antenatal settings in Malawi. These findings support task-sharing models as a means of expanding access to this essential ANC service, particularly in low-resource settings.

**Strengths and limitations of this study**

- The multisite design enhances generalisability across rural, periurban and urban healthcare contexts.
- Large sample size and involvement of the health system stakeholders.
- Absence of a validated tool for assessing scan quality underscores the need for standardised evaluation criteria.
- Incomplete referral data due to the absence of a comprehensive system to accurately track the referrals and referral uptake.

## Introduction

 WHO recommends antenatal ultrasounds (US) before 24 weeks of gestation to help estimate gestational age, enhance the detection of fetal anomalies and multiple pregnancies, minimise labour inductions for post-term pregnancies, and enrich a woman’s pregnancy experience.^1^ Nevertheless, antenatal US is not routinely available in many low-income and middle-income countries (LMICs). This disparity in care may stem from a shortage of US services at antenatal care (ANC) points, a lack of healthcare professionals skilled in US, or unaffordable services for patients, among other reasons.^2^

Since the WHO recommendation, significant advancements have occurred in the portability and affordability of point-of-care US (POCUS). A POCUS device generates images for real-time diagnostic and procedural guidance by the clinician at the point of care, facilitating immediate correlation with signs and symptoms.^2 3^ Research, especially in well-resourced areas, indicates that POCUS can enhance diagnostic accuracy, assist in procedural interventions, shorten diagnostic timeframes and lower overall costs.^4^

Previous research indicates that promoting POCUS use in rural ANC settings can significantly enhance ANC attendance.^5^

In Malawi, access to US services is predominantly limited to tertiary hospitals, typically performed by doctors.^6 7^ The introduction of POCUS can potentially revolutionise ANC, especially given the severe shortage of medical doctors, with a doctor-to-patient ratio of only 0.12 per 1000 people.^6^,^8^ Most women in Malawi receive ANC at primary and secondary health facilities, which are staffed primarily by trained and licensed nurses and midwives.^6 8^ Part-time or full-time clinical officers may occasionally support these facilities. Women with high-risk pregnancies or complications are referred to tertiary facilities, such as district or regional hospitals, where specialised care is provided by obstetrician-gynaecologists, registrars, medical officers and clinical officers.^7^ However, antenatal US is rarely conducted in primary and secondary health facilities in routine practice due to limited equipment and trained personnel.^6^ Consequently, many women give birth without ever undergoing an US, missing critical opportunities to identify maternal and fetal complications.^6 8 9^

This study aimed to address this significant gap by evaluating the feasibility and acceptability of integrating POCUS into routine ANC services. The specific objectives were to describe the feasibility of implementing a novel intervention integrating POCUS into ANC service delivery and to evaluate the acceptability of the intervention among midwives and patients, including in rural and urban health centres. By exploring these objectives, this study seeks to demonstrate the potential of POCUS to enhance ANC delivery in Malawi, improving early detection of maternal and fetal complications and addressing inequities in access to diagnostic technologies.

## Methods

### Conceptual framework

This study draws on implementation science frameworks to guide the assessment of the feasibility and acceptability of introducing the Butterfly iQ POCUS device into ANC settings in Malawi. Specifically, we used the implementation outcomes taxonomy proposed by Proctor *et al*^10^ to structure our data collection and analysis.

We focused on three core implementation outcomes as defined by Proctor *et al*, 2009: (1) Acceptability—stakeholder perception of the intervention as satisfactory; (2) Feasibility—the extent to which the intervention can be carried out in the ANC setting and (3) Appropriateness—the perceived fit or relevance of the intervention for the clinical context. In our study, acceptability was measured by the perceptions among stakeholders that the Butterfly iQ intervention is agreeable, palatable or satisfactory. It reflects users’ direct experiences with the device and related processes (eg, training, workflow integration and quality control (QC)). Unlike general service satisfaction, which reflects broader impressions of service delivery, acceptability was tied specifically to the intervention components. Feasibility captured the extent to which the Butterfly iQ intervention can be successfully used or carried out within ANC settings. This included an evaluation of organisational readiness, provider capacity and infrastructure availability (eg, electricity, connectivity and security). Appropriateness examined whether the Butterfly iQ addressed identified gaps in ANC US services and whether it aligned with national policies, facility priorities and provider scope of practice.

To capture acceptability, we explored users’ experiences with training, satisfaction with the device and app interface, concerns around battery life and durability, and perceptions of the referral process. Feasibility was assessed through questions related to implementation climate, staffing capacity, resource constraints and ANC workflow compatibility. For appropriateness, we examined whether the intervention addressed identified gaps in ANC US service delivery and how it aligned with providers’ professional responsibilities and clients’ expectations.

Our conceptual framework incorporates implementation outcomes and the intervention. The intervention was conceptualised as a broader implementation package that includes the Butterfly iQ device, capacity-building and training for ANC and intrapartum providers; workflow integration strategies; strengthened referral systems and QC and continuous quality improvement mechanisms. This package was developed to ensure alignment with end-user needs and health system structures.

### Study design

This was a two-phase, mixed-method implementation science study conducted across 10 health centres in Blantyre District, Malawi. The study aimed to introduce and integrate POCUS using the Butterfly iQ device into routine ANC delivered by midwives.

- Phase 1 involved theoretical and practical training of midwives followed by a structured mentorship and competency assessment period.
- Phase 2 entailed iterative implementation of the POCUS device alongside routine ANC services and included data collection on feasibility and acceptability through surveys with trained midwives and pregnant women.

### Study setting

The study was conducted in 10 health centres located in both urban and rural areas: Chabvala, Chimembe, Dziwe, Lundu, Mdeka, Limbe, Mbayani, Zingwangwa, South Lunzu and Mpemba. These health centres refer patients to Queen Elizabeth Central Hospital (QECH), the district’s main tertiary hospital. Site selection was done in consultation with the Blantyre District Health Management Office.

### POCUS device

The POCUS device used was the Butterfly iQ, a handheld US probe weighing approximately 313 g and compatible with Apple or Android devices. It connects to a smartphone or tablet through a cable, and the Butterfly iQ app displays images in real time. While Wi-Fi is required for remote teleguidance functions, it is not necessary for routine scanning. Each user was registered with an account through the Butterfly Network.

### Phase 1: training and mentorship

The first phase of the study focused on equipping midwives with the knowledge and skills to perform basic obstetric US scans using the Butterfly iQ device. This phase spanned 5 months and included three sequential components: theoretical training, hands-on demonstrations and a competency-based mentorship programme.

#### Theoretical training (5 days)

Midwives attended a 5-day in-person theoretical training facilitated by eight registrars in their third or fourth year of residency from the Department of Obstetrics and Gynaecology at QECH. Training sessions were delivered using PowerPoint presentations and structured content covering the following topics:

- Basic US physics.
- Safety and infection control protocols.
- Clinical indications for antenatal US.
- Fetal biometry, including measurement of femur length and biparietal diameter.
- Amniotic fluid evaluation.
- Case-based learning using printed scan images and video clips.

#### Hands-on demonstrations

Following classroom instruction, participants were taken to the QECH antenatal ward for practical demonstrations. Under the supervision of registrars, midwives observed real-time scanning of pregnant women across all trimesters. The training illustrated how fetal biometric measures vary by gestational age—for example, the use of crown-rump length in the first trimester and head circumference and femur length in later stages. This exposure allowed midwives to link theoretical knowledge with live clinical contexts and ask clarifying questions during hands-on sessions.

The mentors, whose regular clinical responsibilities include performing obstetric scans, brought substantial practical experience to the demonstrations. These sessions emphasised technique refinement and interpretation of biometric parameters.

### Competency-based mentorship (2+ weeks)

After completing the centralised training, midwives transitioned to mentorship at their respective health centres. Each midwife was paired with a registrar mentor who conducted site visits for individualised, real-time supervision and support. During this period:

- Midwives were required to complete a minimum of 10 US scans using a standardised competency checklist.
- Mentors provided structured, formative feedback during and after each scan.
- Weekly feedback sessions were held to address common challenges and reinforce learning across sites.
- Additional mentorship was extended to midwives who required further support before reaching competency.

**Figure 1:**
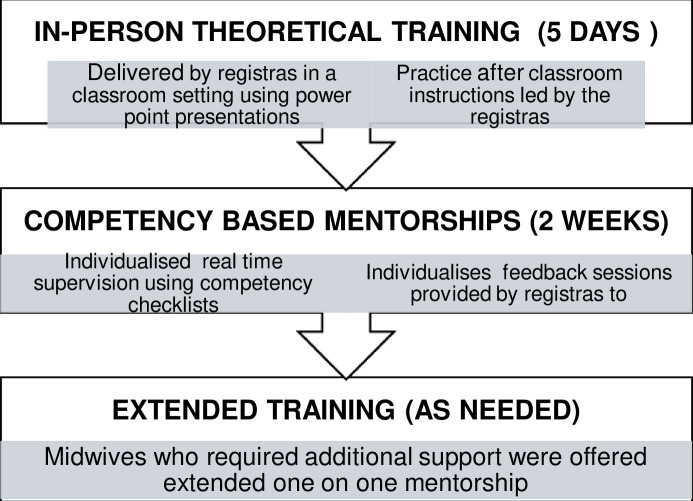
Training components in phase 1 of the study.

Scan quality was monitored through periodic image reviews conducted by registrars. Mentors were temporarily excused from routine hospital duties to prioritise the training, ensuring dedicated guidance throughout the process. Of the 50 midwives who initiated training, 45 successfully completed all three components and were cleared to begin independent scanning in phase 2. Figure 1 summarises the components of training in phase 1.

### Phase 2: implementation and data collection

This phase lasted approximately 8 months. Using stratified sampling across the ten sites, up to 150 pregnant women per site were scanned during their first ANC visit. All were offered a single obstetric scan; those with suspected complications were referred to QECH.

Quality assurance was maintained by periodic reviews of scan images conducted by registrars and follow-up feedback sessions with midwives.

Two sets of surveys were conducted during this phase:

- Trained midwives (n=45).
- Pregnant women scanned during ANC (n=1500).

Surveys were administered by trained research assistants using digital forms on SurveyCTO. Questionnaires were available in English and Chichewa, and interviews lasted approximately 45 min. To assess scan quality, a subset of images was randomly checked by a radiographer and a registrar.

### Note on qualitative evaluation

A separate qualitative evaluation was conducted to explore policy implications and stakeholder perspectives. Findings from this component are being reported in a separate manuscript.

#### Description of counselling

Midwives received training to deliver standard counselling both before and after procedures. Before the procedure, counselling clarified the purpose and importance of scans in ANC. After the procedures, the counselling included results from the scans, such as gestational age and the baby’s position. If complications were detected during the scan, this information was communicated to the pregnant women, and immediate referrals were arranged.

### The intervention

Alongside standard recommended care for pregnant women, midwives incorporated US into routine ANC services.

### Outcome measures

The primary study outcomes were the proportion of midwives trained in basic obstetric scanning using the POCUS device, the number of pregnant women scanned, the feasibility and acceptability of midwife-provided antenatal scanning by midwives and ANC clients, and the proportion of US examinations that met quality standards for a good scan. Secondary outcomes include acceptability-related questions asked of midwives and women.

### Statistical analysis

Participant characteristics and outcome differences (by urban and rural health centres for providers) were assessed using descriptive analysis of survey frequencies and proportions. For categorical variables, we analysed the number and percentage in each category and for continuous variables, we analysed the mean, median, SD, quartiles and range (minimum, maximum).

The proportion of participating health centres offering facilitative care environments for each primary outcome measure is presented. This includes a summary of the number and percentages of ANC clients, midwives and physician participants who reported the study products as feasible and acceptable. Additionally, 95% CIs were calculated for the levels of feasibility and acceptability, using binomial CIs while considering the within-facility correlation of outcomes.

To assess the secondary outcomes, information obtained from questionnaires, in which participants rated acceptability on categorical scales, was used. The number and percentages of ANC clients, midwives and physician participants who reported the study products to be acceptable were summarised. 95% CIs were calculated for the level of acceptability using binomial CIs and considering the within-facility correlation of outcomes.

This study was exploratory and focused on feasibility and acceptability in real-world ANC settings. Therefore, inferential statistical analyses were not conducted. The multisite, descriptive design allowed for capturing practical insights, but future research should incorporate mixed-effects modelling or regression analyses to adjust for potential clustering and confounding variables. This limitation is acknowledged and will guide the design of subsequent implementation and effectiveness studies.

### Patient and public involvement

The study was designed and implemented in collaboration with several important stakeholders, including government policy-makers, service providers and the community. Communities and all stakeholders were actively engaged throughout the duration of the study. Results were also communicated to all the health centres and all the stakeholders inclusive of Blantyre District Health Office and Malawi’s Reproductive Health Directorate.

## Results

### Demographic characteristics of midwives and clients

The study included 45 midwives and 1499 pregnant women who received ANC and US scans. Tables1  2 describe the characteristics of the midwives and the pregnant women respectively. Of the 45 midwives, data from 40 were analysed and disaggregated by facility type (urban or rural). Among these, 47.5% were from rural health centres. The gender distribution included 21 female and 19 male midwives.

**Table 1:** Demographic characteristics of midwives (N=40)

Characteristic	Urban (N=21) N (%)	Rural (N=19) N (%)
Gender		
Female	14 (67)	10 (53)
Male	7 (33)	9 (47)
Qualifications		
Nurse	21 (100)	16 (84)
Midwife	21 (100)	19 (100)
Physician	0 (0)	0 (0)
Years of experience		
Total*	5 (4–9)	5 (2–10)
Providing antenatal care*	5 (3–9)	4 (1–5)
Experience with ultrasound (US)		
Received US as a patient	5 (26)	10 (48)
Observed application of US	11 (58)	12 (57)
Trained on using US	16 (76)	6 (32)
Currently uses US	16 (76)	6 (32)
Household has electricity		
Yes	20 (95)	16 (84)

**Table 2:** Demographics of pregnant women clients (N=1499)

Characteristic	Total (N=1499) N (%)
Location	
Urban	751 (51.8)
Rural	691 (48.2)
Age	
Age in weeks*	25.47 (6.23)
Birth history	
Gravidity*	2 (1–3)
Births (term)*	1 (0–2)
Births (preterm)*	0 (0–0)
Abortions/intrauterine demise*	0 (0–0)
Living children*	1 (0–2)
Current pregnancy	
Gestational age in weeks*	20.07 (5.57)
Estimated trimester	
First	24 (1.7)
Second	399 (27.5)
Third	30 (2.1)
Missing	996 (68.7)
Current symptoms	

	No	Yes
Bleeding	1431 (98.8)	16 (1.1)
Fluid leaking	1440 (99.4)	7 (0.5)
Abdominal pain	1407 (97.1)	40 (2.8)
Headache/visual changes	1381 (95.3)	66 (4.6)
Decreased fetal movement	1432 (98.8)	15 (1.0)
Abnormal urination	1421 (98.1)	26 (1.8)
Abnormal vaginal discharge	1429 (98.6)	18 (1.2)
Other problems	1429 (98.6)	28 (1.9)

* Median (IQR); Numbers may not add to 100% due to rounding.

All midwives were qualified as nurses and midwives, with none holding physician qualifications. The median years of overall clinical experience were similar across urban (5 years, IQR: 4–9) and rural (5 years, IQR: 2–10) settings. Median years of experience in providing ANC were 5 years in urban and 4 years in rural facilities. Prior exposure to US varied: 76% of urban midwives had received training and currently used US, compared with 32% in rural settings. Additionally, a higher proportion of rural midwives (48%) reported having received an US as a patient compared with urban midwives (26%).

Among pregnant women, 51.8% (751/1499) were from urban health centres. The average age was 25.5 years (SD: 6.2), with a median gravidity of 2 (IQR: 1–3) and gestational age around 20 weeks (SD: 5.6). Most women were in their second trimester (27.5%), though trimester data were missing for a majority (68.7%). At the time of visit, 88% of clients reported no danger signs. Only 1.1% reported bleeding, 0.5% fluid leakage, 2.8% abdominal pain and 1.9% other problems. National quintile analysis showed that 36.8% of the clients were from the highest quintile, while 24.9% were from the lowest.

### Feasibility and acceptability of POCUS by midwives

Online supplemental table 1 shows trends of feasibility and acceptability among the midwives. All 40 midwives reported that the Butterfly iQ device was both acceptable and feasible. Furthermore, over 95% indicated they would recommend the device to other midwives. Acceptance of the device’s design and durability was high in both urban (95%) and rural (95%) settings. However, only 52% of all midwives reported that battery life was acceptable, with rural midwives rating it more favorably (68%) than urban ones (38%). This discrepancy may reflect differing operational demands, as urban sites often had higher client volumes and more frequent scanning needs.

The mentorship programme was highly valued by participants, with 100% reporting that it was feasible and helpful. All midwives felt confident in their ability to perform scans independently by the end of the mentorship. Feedback mechanisms within the programme were perceived as helpful by 90% of urban midwives and 100% of rural midwives. When asked about their confidence in the quality of US performed during ANC days, 95% of urban and rural midwives expressed confidence, with only one respondent uncertain.

The likelihood of recommending the device was high, with 76% of midwives giving it a score of 9 or 10 out of 10. Referrals for additional diagnostic scans were relatively low, reported by 20% of midwives overall.

### Routine ANC practice and integration of US 

In Malawi, routine ANC typically follows a four-visit model based on WHO guidelines. While US is not mandatory, it is increasingly recognised as a valuable tool when available. Urban health centres may offer limited sonography through radiographers or physicians, but rural centres often lack equipment or trained personnel. The POCUS intervention introduced midwife-performed scans primarily during the first or second ANC visit to assess gestational age, fetal viability and identify any concerning features (eg, multiple gestation, abnormal presentation). Scans were offered at any point during ANC where midwives deemed them clinically useful, thus integrating US flexibly within existing ANC workflows.

### Client acceptability and feasibility

Women expressed high acceptability for the US experience. Nearly all (99%) felt that receiving an US was important, and 98.5% appreciated having one during the visit. Despite travel and wait times—where nearly half (48.4%) spent more than an hour reaching the clinic and 43.1% waited 30 minutes or more—most women (72.5%) said the time spent on the US was ‘about right’. A majority (57.3%) also said the overall ANC visit duration felt appropriate, while only 6.8% felt it was too short.

In terms of communication, 91.6% of women reported being given the chance to ask questions, and 80.7% were satisfied with how their questions were answered. Nearly all (97.7%) understood why they received the scan, and 98.6% understood the midwife’s explanation of the results. Most women (98.4%) also felt that their privacy was respected throughout the scan.

When asked whether they would recommend the service to others, 76% rated their likelihood at the highest score (10/10), further emphasising strong support and demand for continued use of POCUS in ANC. Table 3 gives an overview of the acceptability and feasibility trends in pregnant women.

**Table 3:** Client acceptability and feasibility of POCUS device (N=1499)

Client acceptability and feasibility measures	Total N=1499
Overall, do you feel your experience with receiving an ultrasound examination today was important?	
No	2 (0.1%)
Yes	1434 (99.0%)
Not sure	13 (0.9%)
How long did it take for you to reach the health centre today?	
Less than 1 hour	702 (48.4%)
1–2 hours	679 (46.9%)
More than 2 hours	68 (4.7%)
How long did you wait before you were seen?	
Less than 30 min	825 (56.9%)
30 min–1 hour	383 (26.4%)
1–2 hours	225 (15.5%)
More than 2 hours	16 (1.1%)
The time spent with the ANC provider during my visit was:	
Too short	99 (6.8%)
Too long	520 (35.9%)
About right	830 (57.3%)
The time spent in my ultrasound was:	
Too short	165 (11.4%)
Too long	233 (16.1%)
About right	1051 (72.5%)
The midwife gave me a chance to ask questions.	
No	121 (8.4%)
Yes	1328 (91.6%)
I am satisfied with the way my questions were answered.	
No	280 (19.3%)
Yes	1169 (80.7%)
I understand why I got my ultrasound.	
No	34 (2.3%)
Ys	1415 (97.7%)
I understood when the midwife explained the results.	
No	21 (1.4%)
Yes	1428 (98.6%)
I appreciated having an ultrasound today.	
No	22 (1.5%)
Yes	1427 (98.5%)
I felt like my privacy was respected before, during and after the ultrasound.	
No	23 (1.6%)
Yes	1426 (98.4%)
How likely are you to recommend this service to someone who needs a similar service?	
1	7 (0.5%)
2	6 (0.4%)
3	1 (0.06)
4	5 (0.3%)
5	29 (2.0%)
6	16 (1.1%)
7	21 (1.4%)
8	118 (8.1%)
9	146 (10.1%)
10	1101 (76.0%)
Total estimated time spent at health centre	
Estimated time in hours*	3.2 (1.3)

* Median (IQR); Numbers may not add to 100% due to rounding.

ANC, antenatal care; POCUS, point-of-care ultrasound scan.

### Quality of scans

Two quality assessments were conducted using independent evaluation tools at different time points by senior registrars in obstetrics and gynaecology. The first assessment took place early in the study, following the initial 8 hours of hands-on US training. At this stage, 50% of scans met the quality standards. The second assessment was conducted after approximately 40 hours of practice, by which point 72% of scans met the required standards. The registrars used a checklist defined in online supplemental table B based on International Society of Ultrasound in Obstetrics and Gynecology (ISUOG) criteria, which included accurate measurements, inclusion of key anatomical views, proper caliper placement and correct labeling.

A third quality review was later conducted by an experienced radiographer after midwives had completed over 120 hours of practice. By this time, most health centres had scanned approximately 150 pregnant women each. According to this assessment, 72% of the scans met the minimum quality standard as defined in online supplemental appendix table A. Table 4 summarises the assessment carried out by the experienced radiographer.

**Table 4:** Ultrasound scan quality assessment by experienced radiographer

Trimester	Total scans	Meets standard—No (%)	Meets standard—Yes (%)
1st	11	4 (36.4)	7 (63.6)
2nd	132	32 (24.2)	100 (75.8)
3rd	7	6 (85.7)	1 (14.3)
Total	150	42 (28.0)***	108 (72.0)***

* Numbers may not total 100% due to rounding.

The differences in outcomes across these assessments reflect both the midwives’ skill acquisition over time and the variability in evaluator criteria. While registrars emphasised diagnostic completeness and clinical relevance, the radiographer focused more on technical image quality.

## Discussion

In this multiphased implementation study, we found it feasible and acceptable to train midwives to conduct POCUS scans as part of routine ANC in Malawi. This aligns with global evidence highlighting the transformative potential of midwife-performed POCUS in low-resource settings.^11 12^ In contexts with limited access to sonographers or radiologists, enabling midwives to offer basic obstetric scans may be a critical step towards reducing preventable maternal and perinatal complications.

Our findings echo experiences from other LMICs. In Ethiopia, the Child Health and Mortality Prevention Surveillance programme demonstrated how midwife training in ultrasonography reduced pregnancy complications and enhanced care-seeking behaviours.^13 14^ Similarly, in Liberia and Uganda, short-duration or phased training programmes equipped midwives with essential POCUS skills, even when they had no prior sonography experience.^15^ Our results confirm that even in a setting like Malawi, where the maternal mortality ratio remains high, midwives can be rapidly trained and supported to deliver high-impact diagnostic services.

A major strength of our intervention was the integrated mentorship model. Midwives who received theoretical training followed by hands-on practice and structured mentoring gained confidence and competence in scan performance. This aligns with literature from Kenya and other sub-Saharan African settings, where mentorship significantly improved skill retention and quality of care.^16^,^18^ By creating ongoing feedback loops between midwives and experienced mentors, our approach contributed to consistent improvements in both self-efficacy and scan quality.

However, high acceptability ratings—especially from ANC clients—should be interpreted with some caution. We acknowledge the potential influence of social desirability bias, particularly given the provider–client relationship and the novelty of the service. Clients may have felt inclined to respond positively to avoid offending providers or to continue receiving perceived high-value care. Although satisfaction was overwhelmingly high (eg, 99% felt US was important, 98.4% felt respected), future studies should consider incorporating anonymous feedback mechanisms or independent assessments to mitigate bias.

Our study also revealed context-specific challenges. In rural facilities, logistical issues such as inconsistent electricity supply, limited internet connectivity and device charging constraints posed barriers to consistent POCUS use. While battery-operated and portable devices such as the Butterfly iQ make it technically feasible to scale US access, infrastructure deficits remain a critical obstacle to routine use in low-resource clinics. Moreover, the cost and sustainability of on-site mentoring limit the scalability of such programmes. Telementoring, while promising, still requires investment in digital tools and training for both mentors and mentees. To enhance sustainability,^19 20^ Malawi and similar contexts may benefit from training midwife-mentors who can provide ongoing support within districts, reducing reliance on external experts.

In addition, the generalisability of our findings should be considered with caution. Malawi’s implementation benefited from an established mentorship network, collaboration with tertiary hospitals and buy-in from district health authorities. These enabling factors may not be present in other countries, especially those with weaker health system infrastructure or limited human resource capacity. Therefore, replication elsewhere would require adaptation to local systems and a deliberate investment in supportive supervision mechanisms.

Our study had several methodological limitations. First, the design was observational and lacked a control group. Health centres were selected purposively rather than randomly, which introduces the risk of selection bias. Without a comparator group, we cannot attribute changes in health outcomes directly to the POCUS intervention. Second, assessments of acceptability and feasibility relied heavily on self-reported measures, which may be susceptible to response bias. Third, while we conducted quality assessments of scans using checklists adapted from international guidelines, we did not employ a formally validated tool such as those used in ISUOG certification processes. Additionally, referral data were incomplete due to the absence of a centralised system for tracking US-related referrals. Finally, we did not compare midwife-performed POCUS with traditional US performed by radiographers or physicians, limiting our ability to comment on diagnostic concordance or clinical outcomes.

Despite these limitations, our study offers several strengths. By capturing the perspectives of both midwives and nearly 1500 pregnant women, we provide a comprehensive picture of implementation feasibility. The findings reflect diverse contexts—urban, periurban and rural—which increases the relevance of our findings for national scale-up. Importantly, the mentor-mentee model not only improved midwife skills but also strengthened referral pathways between primary and tertiary facilities, potentially improving maternal outcomes over time.

### Potential areas for implementation research

Multiple studies have shown that midwives can conduct basic obstetric US scans. US provided by midwives aligns well with the ANC guidelines, which recommend at least one scan before 24 weeks of pregnancy.^19^ However, further research is needed to determine the most effective training model for practising midwives, and health financing studies should explore how to integrate US efficiently and effectively into the ANC at a lower cost.

### Recommendations

- Task sharing was feasible and acceptable, and suggesting further scale-up of the intervention. Scaling-up approaches should not be one-size-fits-all but should cater to all types of midwives, whether in service or part of preservice training. Policy-makers need to establish sustainable methods for providing midwives with in-service training.
- The curriculum for midwives should include basic obstetric US examinations. Training midwives will allow for the practical integration of obstetric US scanning into ANC, which may be more cost-effective than in-service training. The US machines implemented should be suitable for the conditions in LMICs, considering the potential inconsistencies in power supply.
- Collaborations with governments focused on the sustainability of US in ANC are essential, especially regarding additional resources like gel and wipes. The US procedure requires constant resources (examination couch, US gel, etc) to ensure a smooth process. Items such as wipes, gel and beds are necessary for conducting US. Research indicates that integrating US into ANC is feasible; however, beyond just skill, administrative factors also influence the sustainability of US services considered. Moreover, integrating POCUS into existing maternal health policies could further streamline its adoption and enhance sustainability in LMICs.

## Conclusions

In conclusion, this study supports the growing body of evidence that obstetric scanning delivered by midwives using a POCUS device is likely a feasible, acceptable and impactful approach for improving access to scans during routine ANC visits in low-resource settings.

## Supplementary material

10.1136/bmjopen-2025-100515 Supplementary file 1

10.1136/bmjopen-2025-100515 Supplementary file 2

## Data Availability

Data are available on reasonable request.
