# Supplementary material for: Feasibility and acceptability of point-of-care ultrasound delivered by midwives during routine antenatal care in Malawi: a prospective implementation science study
Source: BMJ Open. 2025 Aug 10;15(8):e100515. doi: 10.1136/bmjopen-2025-100515 (PMC12336590; doi:10.1136/bmjopen-2025-100515)
Supplement: Supplementary file 1 [file bmjopen-15-8-s001.docx]

**Appendix A.** Ultrasound items included in the assessment done by the experienced radiographer.

| **Items assessed** | **Response options** |
| --- | --- |
| **First trimester ultrasound** | |
| Presence of gestational sac | Yes / No |
| Size of gestational sac | Yes / No |
| Location of gestational sac | Yes / No |
| Number of gestational sac(s) | Yes / No |
| Presence of a yolk sac | Yes / No |
| Presence of embryo/fetus | Yes / No |
| If embryo/fetus present, assessment of cardiac activity by 2-dimensional video clip or M-mode imaging | Yes / No |
| If embryo/fetus present, was crown rump length measured?* | Yes / No |
| If measured, does measurement appear to have been performed correctly? | Yes / No |
| Examination of uterus | Yes / No |
| Examination of cervix | Yes / No |
| Examination of adnexae* | Yes / No |
| Examination of cul-de-sac region* | Yes / No |
| **Second or third trimester ultrasound** | |
| Fetal number | Yes / No |
| Fetal presentation | Yes / No |
| Amniotic fluid volume | Yes / No |
| Cardiac activity | Yes / No |
| Placental location | Yes / No |
| Fetal biometry | Yes / No |
| Anatomic survey | Yes / No |
| Maternal cervix | Yes / No |
| Maternal adnexae* | Yes / No |
| Other | Yes / No |
| **All ultrasounds** | |
| Did provider adhere to appropriate settings for thermal index and mechanical index?* | Yes / No |
| Did you note any possible errors in obtaining the correct planes for this exam? | Yes / No |
| Did you note any possible errors in obtaining the correct images and/or identification of structures for this exam? | Yes / No |
| Did you note any possible errors in generation of report? | Yes / No |
| Did you note any finding that requires clinical follow-up by the site? | Yes / No |

### Legend:

All items listed were part of the ultrasound quality assessment checklist used by the experienced radiographer. Each item was marked as **“Yes”** if appropriately assessed/documented and **“No”** if not.

- Items marked with an asterisk (*) were **excluded from the final scoring**, as they are not considered part of the core/basic ultrasound skill set for midwives.
- The assessment covered three categories: **first trimester scans, second/third trimester scans,** and **general scan quality across all trimesters.**
- The checklist also included evaluative questions to identify potential **errors in technique or reporting**, as well as any **findings requiring clinical follow-up**.

**Appendix B.** Ultrasound assessment and scoring checklist developed by registrars.

| **Items assessed** | **Response options** |
| --- | --- |
| **First trimester ultrasound scans** | |
| Mid Sac Diameter | |
| Intradecidual sac sign | Yes / No / Not applicable |
| Double decidual sac sign | Yes / No / Not applicable |
| Length/AP/width | Yes / No / Not applicable |
| Crown Rump Midsagittal plane | |
| Focal zone at appropriate level | Yes / No / Not applicable |
| Image magnified appropriately>50% | Yes / No / Not applicable |
| Embryo imaged neutral position | Yes / No / Not applicable |
| Maximum length of embryo shown | Yes / No / Not applicable |
| Max length measured cranial – caudal | Yes / No / Not applicable |
| **Second and third trimester ultrasound scans** | |
| Biparietal Diameter | |
| Focal zone at appropriate level | Yes / No / Not applicable |
| Appropriate magnification 2/3 | Yes / No / Not applicable |
| Axial plane of fetal head | Yes / No / Not applicable |
| Symmetric appearances of hemispheres | Yes / No / Not applicable |
| Midline falx imaged | Yes / No / Not applicable |
| Thalami/ Insula imaged | Yes / No / Not applicable |
| No cerebellum | Yes / No / Not applicable |
| Outside - in caliper | Yes / No / Not applicable |
| Widest diameter | Yes / No / Not applicable |
| Measurement perpendicular to falx | Yes / No / Not applicable |
| Calvarium 75% complete | Yes / No / Not applicable |
| Fetal Length | |
| Focal zone at appropriate level | Yes / No / Not applicable |
| Image magnified appropriately>50% | Yes / No / Not applicable |
| Whole femur diaphysis imaged | Yes / No / Not applicable |
| Ultrasound beam perpendicular long axis of femur | Yes / No / Not applicable |
| Calipers at each end of ossified diaphysis | Yes / No / Not applicable |
| Longest visible diaphysis measured | Yes / No / Not applicable |
| Spur artifacts ant femur ends excluded | Yes / No / Not applicable |
| Amniotic Fluid Index/Deepest Vertical Pocket | |
| Able to measure | Yes / No / Not applicable |
| Placenta location | |
| Able to identify | Yes / No / Not applicable |

**Legend:**

Each ultrasound scan was scored using a checklist comprising key quality indicators. For each item assessed, a score of **1 point** was awarded if the criterion was met. The total score was then calculated for each scan.

- **First trimester scans**: A scan was considered to meet minimum quality standards if it achieved a total score of **≥4.**
- **Second and third trimester scans**: A scan was considered to meet minimum quality standards if it achieved a total score of **≥8.**
