# Supplementary material for: Feasibility and acceptability of point-of-care ultrasound delivered by midwives during routine antenatal care in Malawi: a prospective implementation science study
Source: BMJ Open. 2025 Aug 10;15(8):e100515. doi: 10.1136/bmjopen-2025-100515 (PMC12336590; doi:10.1136/bmjopen-2025-100515)
Supplement: Supplementary file 2 [file bmjopen-15-8-s002.docx]

**Supplementary Table 1: Midwives acceptability and feasibility of POCUS device (N=40).**

| Characteristic | Urban (N=21)  N (%) | Rural (N=19)  N (%) | P-value^ |
| --- | --- | --- | --- |
| **Acceptability** | | | |
| POCUS Device (Overall acceptability) | | | |
| No | 0 | 0 | 1.0 |
| Yes | 21 (100%) | 19 (100%) |  |
| Device (Design) | | | |
| Durability | | | |
| No | 1 (5%) | 1 (5%) | 0.47 |
| Yes | 20 (95%) | 18 (95%) |  |
| Likability of the design | | | |
| No | 0 (0%) | 1 (5%) | 0.47 |
| Yes | 21 (100%) | 18 (95%) |  |
| Easy to use | | | |
| No | 0 (0%) | 1 (5%) | 1.0 |
| Yes | 20 (95%) | 18 (95%) |  |
| Design of web-based application | | | |
| No | 1 (5%) | 0 | 1.0 |
| Yes | 20 (95%) | 19 (100%) |  |
| Have recommended or encouraged other ANC providers to use the device | | | |
| No | 13 (62%) | 1 (5%) | 0.17 |
| Yes | 8 (38%) | 18 (95%) |  |
| Likely to recommend device to another midwife providing ANC service on a scale of 1 to 10 | | | |
| 1 | 0 | 0 |  |
| 2 | 0 | 0 |  |
| 3 | 0 | 0 |  |
| 4 | 0 | 0 |  |
| 5 | 0 | 0 |  |
| 6 | 1 (5%) | 0 |  |
| 7 |  |  |  |
| 8 | 7 (33%) | 6 (32%) |  |
| 9 | 8 (38%) | 3 (16%) |  |
| 10 | 5 (24%) | 10 (53%) |  |
| **Feasibility** | | | |
| Training prepared trainees well to provide ultrasound butterfly iQ | | | |
| Yes | 21 (100%) | 18(95%) | 0.47 |
| No | 0 | 1(5%) |  |
| Participating in the mentoring program was feasible to me | | | |
| Yes | 21 (100%) | 19(100%) | 0.49 |
| No | 0 | 0 |  |
| Mentoring program was helpful to the mentees | | | |
| Yes | 21 (100%) | 19 (100%) | 0.43 |
| No | 0 | 0 |  |
| The feedback received in the program was helpful to me | | | |
| Yes | 19 (90%) | 19 (100%) | 0.42 |
| No | 0 | 0 |  |
| I don’t know | 2 (10%) | 0 |  |
| Overall, think performing ultrasound with Butterfly iQ is feasible | | | |
| Yes | 21 (100%) | 18 (95%) | 0.47 |
| No | 0 | 1 (5%) |  |
| Feel confident about performing obstetric ultrasounds | | | |
| Yes | 21 (100%) | 19 (100%) | 0.42 |
| No | 0 | 0 |  |
| Did you receive referrals for any client’s additional care-based on the ultrasound? | | | |
| Yes | 4 (19%) | 4 (21%) | 1.0 |
| No | 17(81%) | 15(79%) |  |

Legend: ^p-value (fisher’s exact). Numbers may not add to 100% due to rounding. Abbreviations: POCUS – point of care ultrasound; ANC – Antenatal care; iQ -
