# Supplementary material for: Feasibility and acceptability of point-of-care ultrasound delivered by midwives during routine antenatal care in Malawi: a prospective implementation science study
Source: BMJ Open. 2025 Aug 10;15(8):e100515. doi: 10.1136/bmjopen-2025-100515 (PMC12336590; doi:10.1136/bmjopen-2025-100515)
Supplement: Supplementary file 3 [file bmjopen-2025-100515.reviewer_comments.pdf]

PEER REVIEW HISTORY

BMJ Open publishes all reviews undertaken for accepted manuscripts. Reviewers are asked to complete a checklist review form and are provided with free text boxes to elaborate on their assessment. These free text comments are reproduced below.

ARTICLE DETAILS

Title (Provisional)

Feasibility and acceptability of point of care ultrasound delivered by midwives during routine antenatal care in Malawi: a prospective implementation science study

Authors

Payesa, Chipiliro; Seyama, Linly; Chimwaza, Yamikani; Sindani, Fidelis; Kanise, Yankho; Bvutula, Elida; Phiri, Modesta; Nyangulu, Pempho; Gadama, Luis; Kachale, Fanny; Gadama, Gladys; Mwale, Mary; Yenokyan, G; Sripad, Pooja; Hyre, Anne; Noguchi, Lisa M.; Dadabhai, Sufia

---

VERSION 1 - REVIEW

**Reviewer**	**1**
**Name**	**Wittek, Agnes**
**Affiliation**	**University Hospital Bonn**
**Date**	**07-Mar-2025**
**COI**	**None**

This paper highlights a very important topic teaching midwives in ultrasound skills. Although the topic itself is not very new, this topic is highly discussed and proves that midwifes can perform POCUS when trained properly. Additionally the study shows a very high patient satisfaction during their ultrasound examination in pregnancy.

Following I listed some points that need to be improved for publishing this paper:

- Abstract lines 49/50: “Both assessors determined that over 70 of the 350 evaluated scans met the minimum standards for a quality scan.” Please explain or leave out in the abstract. What means “minimum standards” 70 out of 350 is not a high number

- Introduction lines 83-90: Please improve this part leaving out the data from the introduced studies

- Methods:

- Please show three phase training in graphical illustration

- Please include information about theoretical training: how was it performed, what was shown/teached?

- Phase 3 seems to be evaluated in a separate manuscript. Although this is an important information for the reviewer, this information does not need to be included in the manuscript. You may think about leaving out phase 3 and mention that following studies will be performed in the discussion part of the manuscript

- Please descripe the mentorship program in more in detail: How many hours were trained? How many days? How many pictures were taken? How was the feedback system? How much training did the mentors have during their clinical routine work?

- Results:

- Please improve graphical design of the tables

- Table 3: Please mention “can recommend use of Device ANC providers” and explain

- Table 3: “Can recommend device to another midwife on a scale of 1 to 10” Please mention 1 worst, 10 best and explain why only 6,8,9,10 are mentioned

- Lines: 290-295: This part is not really clear. When were the timepoints in the training the professionals perfomed the ultrasound? How many hours of training have been done at first timepoint (50% of satisfying ultrasounds) and second timepoint (72% of satisfying ultrasounds). Please include according to which criteria ultrasound of midwifes was classified as quality standard (eg ISUOG, etc)

- How does a routine ANC in urban vs rural hospitals look like? Is there any timepoint an ultrasound is required in any ANC visit and if so what does this ultrasound include and according to which guideline?

- In general: Expand results part, go more into detail of your data shown in the tables

-

- Discussion: Lacking a control group and other limitations mentioned need to be further discussed. Go more into detail of obstacles performing POCUS in Malawi and give future outlook -> in this case phase 3

- Supplemental: Graphical design of supplemental tables needs to be improved and links to the text need to be included. The yellow lined parts are not clear, please include explanation. Please explain “Fetal Length ≥ 5” or “Biparietal Diameter ≥8”, this is not clear. Maybe include more explanation in the figure legend

For publishing this manuscript mentioned points should be considered and limitations need to be further discussed. Graphical design need to be improved and results should be expanded and more discussed. Please name problems and obstacles of performing ultrasound in Malawi more in detail. Study design of phase one and phase two need to be expanded.

**Reviewer**	**2**
**Name**	**Recker, Florian**
**Affiliation**	**University of Bonn**
**Date**	**08-Apr-2025**
**COI**	**None**

This manuscript titled "Feasibility and acceptability of point-of-care ultrasound (POCUS) services delivered by midwives during routine antenatal care in Malawi" addresses a highly relevant and timely topic in maternal healthcare delivery in low- and middle-income countries (LMICs). The study evaluates the implementation of POCUS by trained midwives as part of routine antenatal care (ANC), responding directly to WHO guidelines and filling an important gap in both access and task-sharing strategies. The manuscript benefits from a strong participatory design, a large sample size, and involvement of both community and health system stakeholders. However, substantial revisions are needed before the manuscript is suitable for publication.

The structure and presentation of the study phases need considerable clarification. While the authors describe a three-phase implementation model, the narrative around these phases is inconsistent and at times confusing—particularly regarding Phase 3, which involves qualitative data that are mentioned but not reported in this manuscript. If Phase 3 findings are to be reported elsewhere, this should be clearly stated and references to it in the current text should be minimized or reframed appropriately.

From a methodological standpoint, the study relies heavily on descriptive statistics without including inferential analyses that would strengthen the findings. Given the multi-site nature of the study and the availability of data from both rural and urban centers, more rigorous

statistical analysis (e.g., adjusted models accounting for clustering or confounding variables) would improve the scientific value of the manuscript. If inferential analyses were not conducted due to limitations, this should be explicitly stated and justified.

Another major concern is the assessment of scan quality, which is central to evaluating feasibility. While the manuscript reports the proportion of scans that met “minimum standards,” it is not always clear how these standards were defined or operationalized. Furthermore, the discrepancy between the evaluations by radiographers and registrars (e.g., initial quality rates as low as 50%) warrants further discussion. Detailed explanation of the scoring tools used, the thresholds for “acceptable” quality, and their relevance to clinical safety and diagnostic accuracy should be provided.

The manuscript also uses key terms such as “feasibility,” “acceptability,” and “satisfaction” somewhat interchangeably. These concepts should be defined early in the methods section and used consistently throughout the manuscript. Similarly, sections of the text contain redundant phrases and would benefit from careful language editing to improve clarity and coherence. Several grammatical issues and awkward constructions also reduce the readability of the manuscript and should be addressed through thorough proofreading.

An important limitation of the study is the lack of detailed reporting on adverse events, referrals, and outcomes following ultrasound exams. While referrals to higher-level facilities are mentioned, the manuscript does not offer a clear picture of how many patients were referred, for what reasons, and what happened subsequently. Even if such data are not available, the absence should be acknowledged as a limitation, particularly since patient safety is a key consideration in scaling POCUS interventions.

In the discussion section, the authors highlight the promise of midwife-performed POCUS and cite similar studies from Ethiopia, Liberia, and Uganda, which is commendable. However, the interpretation of the findings could be deepened. For example, while high satisfaction rates are reported, the possibility of social desirability bias—particularly given the provider–client relationship—should be addressed more thoroughly. Likewise, the generalizability of findings to other contexts (e.g., countries without the same training or mentorship infrastructure) should be discussed in greater depth.

Despite these concerns, the study presents a compelling case for task-sharing in antenatal ultrasound delivery and demonstrates the acceptability of POCUS among both providers and clients in a real-world setting. The mentorship model employed appears particularly promising and aligns with current global recommendations for workforce strengthening in maternal health.

In conclusion, while the manuscript makes an important contribution to the literature, it requires substantial revisions to improve its scientific rigor, presentation, and interpretation of findings. I recommend a decision of major revision. Specifically, the authors should: clarify the study phases and remove or minimize references to data not reported in this manuscript; expand and justify their analytic methods; provide more detail on scan quality

assessment; address potential biases and limitations more explicitly; and revise the language and structure for clarity. With these improvements, the manuscript has strong potential to inform ANC policy and practice in LMICs.

VERSION 1 - AUTHOR RESPONSE

Response to Reviewer 1 Comments (Dr. Agnes Wittek)

Comment:
Abstract lines 49/50: "Both assessors determined that over 70 of the 350 evaluated scans met the minimum standards for a quality scan." Please explain or leave out in the abstract. What means "minimum standards" 70 out of 350 is not a high number

Response:
Thank you for this comment. We have revised the sentence in the abstract to clarify that '70%' (not 70 scans) of the 350 evaluated scans met minimum standards. We also define what constitutes 'minimum standards' in the Methods section, referencing ISUOG-based criteria.

Comment:
Introduction lines 83-90: Please improve this part leaving out the data from the introduced studies

Response:
We have revised this section to remove overly detailed data from other studies, focusing instead on setting the context and rationale for our study.

Comment:
Methods:
- Please show three phase training in graphical illustration
- Please include information about theoretical training: how was it performed, what was shown/teached?
- Phase 3 seems to be evaluated in a separate manuscript. Although this is an important information for the reviewer, this information does not need to be included in the manuscript. You may think about leaving out phase 3 and mention that following studies will be performed in the discussion part of the manuscript
- Please descripe the mentorship program in more in detail: How many hours were trained? How many days? How many pictures were taken? How was the feedback system? How much training did the mentors have during their clinical routine work?

Response:
Thank you for these detailed suggestions. We have: (1) included a new figure to illustrate the three-phase training and evaluation process; (2) expanded the Methods section to describe the theoretical training, including lectures, and hands-on practise;

(3) removed Phase 3 from the Results. and (4) described the mentorship program in detail.

Comment:
Results:
- Please improve graphical design of the tables
- Table 3: Please mention "can recommend use of Device ANC providers" and explain
- Table 3: "Can recommend device to another midwife on a scale of 1 to 10" Please mention 1 worst, 10 best and explain why only 6,8,9,10 are mentioned
- Lines: 290-295: This part is not really clear. When were the timepoints in the training the professionals perfomed the ultrasound? How many hours of training have been done at first timepoint (50% of satisfying ultrasounds) and second timepoint (72% of satisfying ultrasounds). Please include according to which criteria ultrasound of midwifes was classified as quality standard (eg ISUOG, etc)
- How does a routine ANC in urban vs rural hospitals look like? Is there any timepoint an ultrasound is required in any ANC visit and if so what does this ultrasound include and according to which guideline?
- In general: Expand results part, go more into detail of your data shown in the tables

Response:
We have implemented the following changes: (1) all tables have been reformatted for clarity; (2) Table 3 has been updated to clarify question wording, rating scale (1=worst, 10=best), and inclusion of all relevant response categories; (3) we clarified timing and duration of ultrasound performance at each timepoint—2 weeks and 4 weeks into training respectively, with corresponding training hours; (4) scan quality classification was based on ISUOG-adapted criteria; and (5) a subsection now discusses urban vs. rural ANC settings and existing national ultrasound guidelines. We also expanded the Results section to provide greater detail from the data tables.

Comment:
Discussion: Lacking a control group and other limitations mentioned need to be further discussed. Go more into detail of obstacles performing POCUS in Malawi and give future outlook -> in this case phase 3

Response:
We have expanded the Discussion to more thoroughly address the lack of a control group, power supply issues, staff shortages, and other operational barriers..

Comment:
Supplemental: Graphical design of supplemental tables needs to be improved and links to the text need to be included. The yellow lined parts are not clear, please include explanation. Please explain "Fetal Length ≥ 5" or "Biparietal Diameter ≥8", this is not clear. Maybe include more explanation in the figure legend

Response:
We have improved the formatting of supplemental tables and added clear links between tables and the main text. Figure legends now explain all parameters, including 'Fetal Length and 'Biparietal Diameter

Response to Reviewer 2 Comments (Dr. Florian Recker)

Comment:
The structure and presentation of the study phases need considerable clarification. While the authors describe a three-phase implementation model, the narrative around these phases is inconsistent and at times confusing—particularly regarding Phase 3, which involves qualitative data that are mentioned but not reported in this manuscript. If Phase 3 findings are to be reported elsewhere, this should be clearly stated and references to it in the current text should be minimized or reframed appropriately.

Response:
We have revised the manuscript to minimize references to Phase 3. We have clarified that the qualitative data will be presented in a separate manuscript.

Comment:
From a methodological standpoint, the study relies heavily on descriptive statistics without including inferential analyses that would strengthen the findings. Given the multi-site nature of the study and the availability of data from both rural and urban centers, more rigorous statistical analysis (e.g., adjusted models accounting for clustering or confounding variables) would improve the scientific value of the manuscript. If inferential analyses were not conducted due to limitations, this should be explicitly stated and justified.

Response:
Thank you for this feedback. As this is an implementation study, we focused on descriptive statistics to evaluate feasibility and acceptability. We now explicitly justify this in the manuscript and note the potential for future inferential analysis.

Comment:
Another major concern is the assessment of scan quality, which is central to evaluating feasibility. While the manuscript reports the proportion of scans that met “minimum standards,” it is not always clear how these standards were defined or operationalized. Furthermore, the discrepancy between the evaluations by radiographers and registrars (e.g., initial quality rates as low as 50%) warrants further discussion. Detailed explanation of the scoring tools used, the thresholds for “acceptable” quality, and their relevance to clinical safety and diagnostic accuracy should be provided.

Response:
We have revised the Methods to define scan quality clearly using ISUOG-based standards. We also describe the assessment tools, the criteria for acceptability, and the differences in emphasis between registrar and radiographer evaluations.

Comment:
The manuscript also uses key terms such as “feasibility,” “acceptability,” and “satisfaction” somewhat interchangeably. These concepts should be defined early in the methods section and used consistently throughout the manuscript.

Response:
We have now defined feasibility, acceptability, and satisfaction in the Methods using established frameworks and ensured consistent usage throughout the manuscript.

Comment:
Similarly, sections of the text contain redundant phrases and would benefit from careful language editing to improve clarity and coherence. Several grammatical issues and awkward constructions also reduce the readability of the manuscript and should be addressed through thorough proofreading.

Response:
Thank you. The manuscript has been thoroughly proofread and revised to eliminate redundancies, clarify language, and correct grammar.

Comment:
An important limitation of the study is the lack of detailed reporting on adverse events, referrals, and outcomes following ultrasound exams. While referrals to higher-level facilities are mentioned, the manuscript does not offer a clear picture of how many patients were referred, for what reasons, and what happened subsequently. Even if such data are not available, the absence should be acknowledged as a limitation, particularly since patient safety is a key consideration in scaling POCUS interventions.

Response:
We have added a note in the Limitations section acknowledging the absence of detailed referral and outcome data and recommending this be included in future evaluations.

Comment:
In the discussion section, the authors highlight the promise of midwife-performed POCUS and cite similar studies from Ethiopia, Liberia, and Uganda, which is commendable. However, the interpretation of the findings could be deepened. For example, while high satisfaction rates are reported, the possibility of social desirability bias—particularly given the provider–client relationship—should be addressed more thoroughly. Likewise, the generalizability of findings to other

contexts (e.g., countries without the same training or mentorship infrastructure) should be discussed in greater depth.

Response:
We have revised the Discussion to address potential social desirability bias and reflected on how the training and mentorship infrastructure may influence generalizability.
